# Mapping the landscape of land inequality: A multi-level, data-driven exploration of land inequality in South Korea’s urban and regional spheres

**DOI:** 10.1371/journal.pone.0320252

**Published:** 2025-03-25

**Authors:** Yookyung Lee, Seungwoo Han

**Affiliations:** 1 Yongin Research Institute, Yongin, South Korea; 2 Kyonggi University, Suwon, South Korea; University of Innsbruck: Universitat Innsbruck, AUSTRIA

## Abstract

Land inequality has emerged as a defining challenge in contemporary societies, shaping patterns of wealth concentration and socioeconomic stratification. This study critically examines the persistence and intensification of land disparities in South Korea between 2018 and 2022, applying a data-driven methodological framework to uncover structural inequalities embedded within the spatial distribution of land wealth. The analysis reveals a stark divergence in property values and ownership patterns, reinforcing existing socioeconomic divisions and highlighting the spatial entrenchment of privilege. The implications of these disparities extend beyond economic inequities, raising concerns about long-term social stability and policy effectiveness in mitigating wealth concentration. This study underscores the necessity of policy interventions to address the compounding effects of land inequality. By advancing an empirical approach to the study of land concentration, this research contributes to broader discussions on spatial inequality, economic polarization, and sustainable urban development.

## Introduction

Land, as a finite and irreplaceable resource, serves as a fundamental pillar in shaping economic structures, societal hierarchies, and power relations. Its significance extends beyond its physical attributes of soil and terrain; rather, land derives its value primarily from its location. The equitable distribution of land is a critical determinant of social stability and economic prosperity, yet land inequality remains a persistent and deepening global challenge [1,2]. This disparity manifests in multiple dimensions, including ownership concentration, accessibility to land-based resources, and spatial inequalities, all of which have profound implications for socioeconomic development [3–5] and social cohesion [6–9]. In the modern era, the effects of land inequality are particularly pronounced in rapidly urbanizing regions, where the concentration of wealth, power, and opportunities exacerbates tensions and disparities [10]. The complex interplay between land, power, and wealth has thus become a central focus of inquiry, revealing the diverse impacts of land inequality on social sustainability and cohesion.

In this broader global context, South Korea (hereafter referred to as Korea) provides a compelling case study of land inequality’s cyclical nature. Once regarded as a model of equitable development due to its successful post-independence land reforms, Korea is now witnessing a resurgence of extreme land inequality. In the pre-modern era, the country faced severe land concentration, which was significantly mitigated through state-led redistribution policies after liberation. However, in recent decades, soaring real estate values and wealth polarization have once again deepened disparities in land ownership.

This phenomenon is particularly evident in the Seoul Metropolitan Area, where the high concentration of economic and social activities intensifies the effects of land inequality, making the region a reflective case of broader national and global challenges [11]. Historical factors, such as Seoul-centralized government policies aimed at national development [12], combined with transformative events like the 1997 Asian financial crisis [13,14], have exacerbated these inequalities.

While existing literature provides valuable insights into regional inequality [11,15–18], research on the inter-regional and intra-regional dimensions of land inequality remains limited, particularly in terms of its spatial and temporal evolution in Korea. This study investigates the evolution of land inequality in Korea from 2018 to 2022, focusing on both inter-regional and intra-regional disparities. By analyzing spatial patterns in land wealth distribution, this research seeks to identify structural inequalities and their persistence over time. Through this comprehensive investigation, this study aims to provide a deeper understanding of the spatial concentration of land wealth and its broader socioeconomic implications.

To achieve these objectives, this study adopts a data-driven analytical framework that examines inter-regional and intra-regional land inequality. Inter-regional inequality is assessed through empirical land price metrics, specifically land price per square meter, which captures disparities in property values across different districts. In contrast, intra-regional inequality is measured using indicators of wealth concentration within individual districts, highlighting disparities among households in the same locality. By integrating these complementary dimensions, this study provides a multi-layered perspective on Korea’s evolving land inequality.

The methodological approach combines data visualization techniques for an intuitive representation of spatial disparities with machine learning algorithms—specifically clustering techniques—to identify structural patterns in land inequality. The clustering results are further validated using silhouette analysis to ensure the robustness of segmentation and enhance the interpretability of findings. This analytical framework allows for a systematic investigation of the spatial concentration of land wealth and its broader socioeconomic implications.

The findings reveal an entrenched pattern of land inequality, with a bifurcation into two distinct clusters representing differential levels of land inequality. This division underscores significant disparities in land values, particularly in districts such as Gangnam-gu and Seocho-gu, which have emerged as centers of concentrated affluence. Moreover, from 2018 to 2022, intra-regional land inequality has intensified, reflecting a growing accumulation of land wealth among a limited segment of the population. These findings suggest that land inequality in Korea is not only a persistent issue but also one that is structurally embedded and intensifying over time.

The implications of this research are significant for urban planners, policymakers, and social scientists seeking to design interventions aimed at mitigating land-based disparities. A nuanced understanding of inter- and intra-regional inequality patterns can inform more equitable land-use policies and development strategies. Furthermore, by demonstrating the utility of data-driven methodologies, this study contributes to advancing empirical research on land inequality, offering insights that can inform both academic discourse and policy frameworks.

This study contributes to the literature by employing a data-driven approach to examine inter- and intra-regional land inequality in Korea, offering critical insights into the structural underpinnings of these disparities. Recent scholarship has increasingly emphasized the role of asset inequality—particularly disparities in land prices and ownership—in shaping various societal outcomes, including public policy preferences, political behavior [19–26], and overall quality of life [27–31]. By providing empirical evidence on the persistence and intensification of structural land inequality, this study reinforces and extends these discussions. In doing so, it not only substantiates prior research but also offers a more nuanced perspective that can inform future academic inquiry and policy deliberations aimed at addressing spatial and economic disparities.

### Land inequality and social dynamics

#### Land as a catalyst for inequality: the societal implications of location and ownership.

The relationship between land and inequality has been a central theme in economic and social discourse, particularly in the context of societal progress and development. In his seminal work, George [32] highlights the persistent nature of poverty and economic disparity, arguing that these challenges are fundamentally tied to land—not merely as a physical resource but as a location-based asset whose value is intrinsically linked to accessibility and desirability. As societies grow and urbanization intensifies, land scarcity becomes more pronounced, and its value is increasingly dictated by its proximity to economic hubs, educational institutions, transportation networks, and other essential resources. This locational advantage, rather than the inherent characteristics of land itself, becomes the primary determinant of wealth accumulation and socioeconomic stratification.

Building upon George’s foundational argument, contemporary scholars have expanded the discussion to explore how land value and ownership intersect with broader structural inequalities. Smith [33], for instance, introduces the concept of “friction of distance,” which suggests that physical proximity to high-value amenities confers significant social and economic advantages, reinforcing disparities in access to resources. This framework highlights how spatial arrangements within urban environments create differentiated opportunities, often privileging certain populations while marginalizing others. This uneven distribution of locational advantages manifests in patterns of exclusion and economic segmentation, where those who own land in prime locations accrue substantial benefits, further widening the socioeconomic gap.

Recent scholarship further underscores that land value is shaped by the social and economic advantages embedded within specific locations [34–37]. Access to high-quality infrastructure, superior public services, and economic opportunities significantly elevates land prices, reinforcing spatial hierarchies and wealth concentration. In this context, real estate functions as a *positional good*, aligning with Veblen’s [38] theory of status-driven consumption, where ownership signals economic privilege and social distinction. Beyond its role as a financial asset, property ownership serves as a mechanism for intergenerational wealth transfer, enabling landowners to consolidate economic power while limiting opportunities for non-owners to enter high-value property markets [11,15–18]

The increasing competition for land in desirable locations has intensified these disparities, erecting systemic barriers to social mobility. The lack of access to high-value properties disproportionately affects lower-income groups, further entrenching economic stratification. This dynamic is particularly evident in Korea’s urban real estate markets, where escalating property values in prime locations exacerbate exclusionary patterns, limiting access to housing, quality education, and employment prospects [11,15–18].

This expanding body of research highlights that disparities in land ownership and valuation are not merely economic concerns but fundamental indicators of broader structural inequalities. The widening gap in land values not only reflects growing economic polarization but also deepens disparities in social engagement and political participation [31,39]. As land ownership increasingly dictates access to essential resources and opportunities, it functions both as a product of existing inequalities and as a driving force that perpetuates them.

Recognizing the critical role of land in shaping social hierarchies, scholars from diverse disciplines have examined its influence on political attitudes and policy preferences [19–22,24–26,40]. Research suggests that land ownership is associated with distinct political behaviors, particularly in relation to property taxation, urban development policies, and regulatory frameworks. Additionally, other studies highlight the psychological and emotional dimensions of real estate ownership, emphasizing the security, stability, and sense of belonging that property ownership affords individuals and families [27–31,41]. These findings indicate that land inequality has profound consequences beyond economic stratification, shaping individual well-being, community cohesion, and political engagement.

Given these multidimensional implications, it is crucial to approach land inequality as a deeply embedded structural issue with far-reaching effects. Understanding its historical evolution, spatial manifestations, and socioeconomic consequences enables a more comprehensive analysis of its role in perpetuating broader societal disparities. Whether individuals own land or not significantly influences their economic prospects, social standing, and political agency. As such, addressing land inequality requires not only economic interventions but also policy measures that acknowledge its intersection with social and political inequalities. This perspective shifts the discourse on land from a purely economic asset to a fundamental determinant of social stratification, reinforcing the urgency of addressing its unequal distribution in contemporary societies.

#### Land inequality in the context of Korea.

The successful implementation of land reform in Korea after its liberation from Japanese colonial rule in 1945 exemplifies how targeted policies can accelerate economic development [42–47]. At the time, Korea was predominantly an agrarian society, with 77% of the population engaged in farming [46]. The land reforms aimed to dismantle a system dominated by large landholdings and tenant farming, a legacy of pre-modern structures and Japanese colonialism.

In 1945, only 14% of farming households owned the land they worked, while tenants managed 65% of all agricultural land, including 71% of rice fields [46]. Addressing this deep-seated inequality was essential for Korea’s transition to independence. The agrarian reform is often credited as a key factor in Korea’s rapid economic growth [42–44,46,47], redistributing land and laying the groundwork for a more equitable economy. This reform preceded Korea’s rapid industrialization in the 1970s and democratization in the 1980s, positioning it as a model for development in Asia [48].

Following the 1997 Asian Financial Crisis, Korea’s economic structure shifted, with a new emphasis on assets and capital over wages and income [13,14]. This transition has exacerbated economic inequalities and stratification. Oh [13] links these disparities to inflation in asset values, particularly in real estate. Weon and Rothwell [49] further highlight that homeownership reduces the risk of asset poverty, indicating that those without assets face systemic poverty. Additionally, Seoul-centric policies during the 1970s and 1980s contributed to regional wealth disparities [12].

The issue of real estate inequality emerges as a prominent challenge within Korean society, exemplified strikingly by a contentious 2023 advertisement for a residential complex in Banpo-dong, Seocho-gu, Seoul. The advertisement, which provocatively stated, “We’ve built this for those who’ve always dreamt of an unequal world,” not only underscores the elevated societal valuation of property ownership but also blatantly reflects classist sentiments [49].

In Korea’s sociocultural context, geographical locations carry significant symbolic weight. This phenomenon is illustrated through the stark contrasts between metropolitan Seoul and its rural counterparts, the delineation between areas within and outside Seoul, and the distinctions between regions such as Gangnam and Gangbuk [50–52]. These geographical distinctions act as markers of personal identity, social status, and economic class [11,53,54]. The symbolic value attributed to real estate is profound, often metaphorically referred to as the “Republic of real estate.” This perception may be deeply ingrained in the cultural heritage of East Asia’s agrarian societies, where the historical importance of land, particularly for rice cultivation, was paramount, a concept explored by Talhelm et al. [55]. Furthermore, the historical development of Gangnam during the rapid economic growth and real estate boom of the 1970s and 1980s played a pivotal role in reinforcing the significance of real estate in shaping societal values and aspirations, as analyzed by Yang [52].

**Fig. 1:**
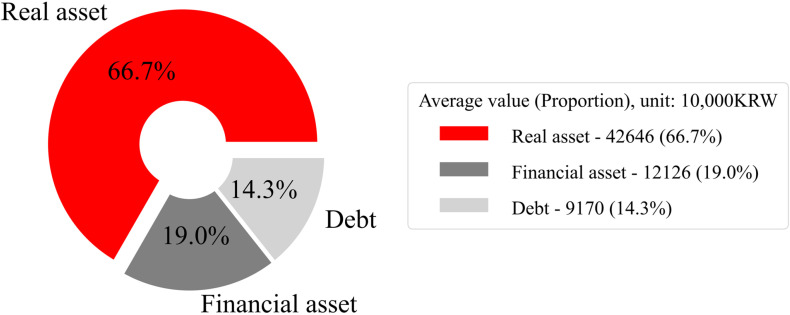
Household asset composition KRW refers to the Korean Won, and as of August 30, 2024, 1 dollar is equivalent to 1,325.5 KRW (https://stat.molit.go.kr/portal/main/portalMain.do). Source: K·indicator (https://www.index.go.kr/unify/idx-info.do?idxCd=8087).

Fig 1 offers a comprehensive analysis of asset distribution among Korean households, underscoring a significant bias towards real assets. Specifically, the data indicates that an average of 66.7% of an individual’s total assets in Korea are in the form of real assets, most prominently real estate. In contrast, financial assets constitute a mere 19% of the aggregate asset portfolio. This high ratio of real assets is conspicuously elevated in comparison with other member states of the Organisation for Economic Co-operation and Development [OECD] [56].

The high proportion of real assets in Korea, particularly land, is notably elevated compared to other OECD member states. According to OECD data [56], land constitutes a significant share of non-financial assets in Korea, with non-produced assets, including land, accounting for 53% of total non-financial assets. This figure surpasses that of countries such as France (46%), Japan (42%), and Canada (35%), highlighting the extent to which land wealth dominates Korea’s economic landscape. These statistics suggest that a substantial fraction of Korean wealth is invested in real estate.

**Fig. 2:**
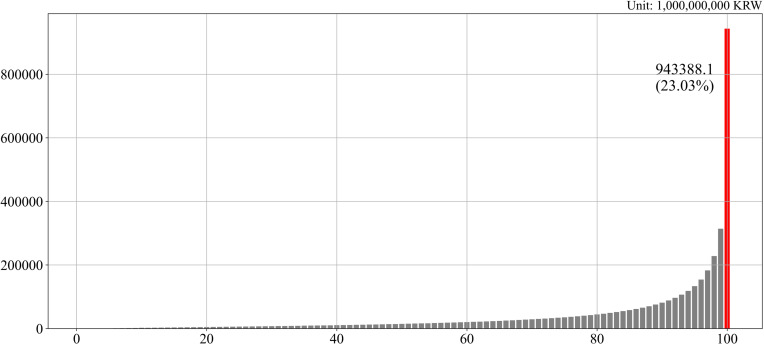
Total value of land at the 100th percentile (2022). Source: Ministry of Land, Infrastructure and Transport.

Fig 2 presents a detailed analysis of the concentration of land wealth among Korean households, highlighting the extent of inequality in land ownership. The data indicate that the top 1% of households, measured by total land value, collectively hold 23.03% of all privately owned land in Korea. In contrast, households in the bottom 40% of the distribution own only 4.97% of the total land value. The disparity becomes even more pronounced when examining the top 10% of households, which together control 57.76% of the nation’s privately owned land. In other words, the aggregate land value held by the top decile surpasses that of the remaining 90% combined, underscoring the extent to which land wealth is concentrated among a small segment of the population. This pattern reflects a highly skewed distribution of land assets, reinforcing broader concerns about structural inequality and wealth consolidation in Korea.

**Fig. 3:**
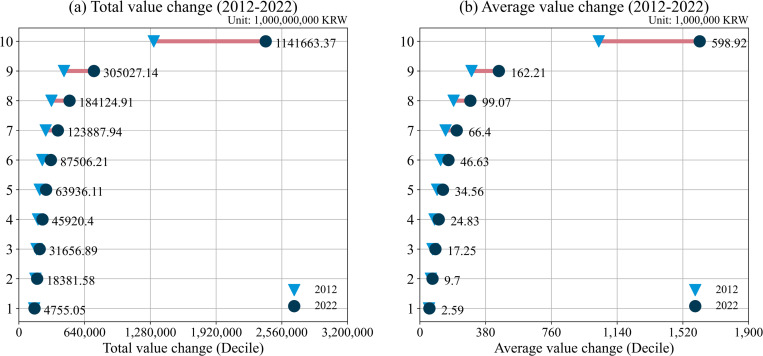
Total value and average value of land (1th to 10th). *Note*: The numbers in the plots represent the increase from 2012 to 2022.Source: Ministry of Land, Infrastructure and Transport (https://stat.molit.go.kr/portal/main/portalMain.do).

Fig 3(a) presents a longitudinal analysis of the changes in land values across household deciles in Korea from 2012 to 2022. The data reveal a dramatic increase, with households in the 10th decile experiencing more than a twofold rise in the total value of their land over the decade. Furthermore, the data illustrate a widening disparity in the absolute value of land ownership among different subgroups over this ten-year span. Fig 3(b) complements this analysis by examining the average land values across deciles, from the 1st to the 10th, and their respective changes between 2012 and 2022. Consistent with Fig 3(a), the 10th decile witnessed a significant increase in average land value, outstripping growth in other deciles. As of 2022, households in the 10th decile, on average, possess land valued at approximately four times that of households in the 9th decile, and an astonishing 244 times greater than that of households in the 1st decile. Taken together, these figures underscore the widening inequality in real estate ownership within Korea. The observed trends point to a deepening economic divide, particularly manifested in the realm of land ownership.

## Materials and methods

### Data-driven methods and data

This study adopts a data-driven approach to quantify land ownership disparities in Korea, using data not merely as numerical inputs but as tools to reveal underlying social dynamics, as emphasized by Monroe et al. [57]. By doing so, we seek to uncover the social relationships driving land inequality and illustrate how these relationships manifest across spatial configurations. Rather than comparing clustering algorithms, we select the most appropriate method based on research objectives, data characteristics, and contextual factors, as recommended by von Luxburg et al. [58], Henning and Liao [59], and Hennig [60]. This approach prioritizes data-driven insights over model-driven analyses.

The primary aim is to examine both inter- and intra-regional land ownership disparities by analyzing spatial patterns, providing a deeper understanding of the social dynamics underlying asset inequalities in Korean society. A critical aspect of this approach is the careful selection of indicators aligned with the study’s objectives. As Harvey [61] notes, variables used to analyze inequality should reflect the specific societal context. In this case, the focus is on measuring land price and ownership disparities to capture the dimensions of inequality in Korea.

Table 1 presents the key variables utilized in this study, derived from raw data provided by the Ministry of Land, Infrastructure, and Transport and refined to align with the study’s objectives (see Appendix A). The analysis specifically focuses on land assets owned by individual households, deliberately excluding public and corporate holdings to ensure a more precise examination of inter-regional and intra-regional land inequality for the years 2018 and 2022. Given that detailed district-level data from the Ministry of Land, Infrastructure, and Transport is available from 2018 onwards, this timeframe allows for a systematic temporal analysis of evolving disparities.

**Table 1:** List of variables.

	Variable	Description
1	Land price/m2	This variable is the price per square meter of land and refers to the difference in land prices between districts (*si-gun-gu*), i.e., inter-regional land inequality.
2	Intra-land inequality	This variable represents the ratio within the district (*si-gun-gu*) level, where the numerator is the number of households with land worth more than a billion KRW, and the denominator is the number of households with land worth less than fifty million KRW, i.e., intra-regional land inequality.

*Note*: See Appendix for details.

By concentrating on household-level data, this study emphasizes disparities in personal wealth as reflected in land ownership, a critical determinant of socioeconomic status. This approach enables a more granular investigation into how land assets are distributed across different socioeconomic groups, providing empirical insights into the structural patterns of inequality. By integrating this household-focused perspective with a broader data-driven analysis, this study enhances the understanding of land inequality’s spatial and economic dimensions in Korea.

To assess inter-regional land inequality, this study utilizes land price per square meter (KRW/m²) as the primary metric, which provides a standardized measure of land value disparities across districts (si-gun-gu). This variable offers an empirically grounded approach to comparing structural inequalities at the regional level, as differences in land prices across districts reflect spatial concentration patterns and the uneven distribution of economic opportunities.

For intra-regional land inequality, a ratio-based indicator is employed, capturing the distribution of land ownership within each district rather than absolute price levels. Specifically, this metric is calculated as the number of households with land assets exceeding one billion KRW divided by the number of households with land assets below fifty million KRW. This approach is adapted from the Palma ratio, commonly used to measure income inequality, by comparing the top 10% of landowners to the bottom 40%. The rationale behind using this ratio-based measure, rather than price per square meter, is that land price alone does not fully capture wealth disparities within a single district. By focusing on ownership concentration at the two ends of the wealth spectrum, this indicator provides a more precise measure of intra-regional inequality in land holdings.

To illustrate, data from Statistics Korea indicate that households in the 4th decile (bottom 40%) have an average land asset value of 49.7 million KRW, while those in the 10th decile (top 10%) hold an average of 1.005 billion KRW in land assets. The ratio-based measure thus enables a comparative assessment of how land wealth is distributed among different socioeconomic groups within each district.

In summary, the distinction in measurement units—KRW/m² for inter-regional inequality and a ratio for intra-regional inequality—is driven by the need to appropriately capture disparities at different spatial scales. Land price per square meter provides a direct comparison of regional land values, whereas the ratio-based approach accounts for wealth concentration within individual districts, where absolute price comparisons may not fully reflect disparities in ownership distribution.

### Analysis strategy

This study employs a computational approach to analyze land inequality in Korea, utilizing data-driven methodologies to examine both inter-regional and intra-regional disparities. To systematically classify regions based on their land inequality metrics, the study applies the K-means++ clustering algorithm, which is chosen for its ability to optimize initial centroid selection, thereby improving clustering accuracy and robustness [62]. This method enables the identification of distinct regional patterns in land inequality, facilitating a more structured analysis of spatial disparities in land ownership and valuation.

To validate the clustering results and ensure the most appropriate segmentation, the silhouette score is employed as an evaluative metric. This measure assesses the cohesion within clusters and the separation between them, providing an objective criterion for determining the optimal number of clusters [63]. The analysis is further refined through silhouette plots, which visually represent the quality of clustering and confirm the stability of the selected cluster configuration.

By integrating machine learning techniques with data visualization, this study offers a multi-dimensional perspective on land inequality, capturing both regional concentration patterns and intra-regional ownership disparities. This methodological framework enhances the precision of inequality measurement and provides a structured approach to understanding the spatial manifestations of land wealth concentration in Korea. Further details on data preprocessing, and clustering implementation are elaborated in the subsequent sections.

## Results of analysis

**Fig. 4:**
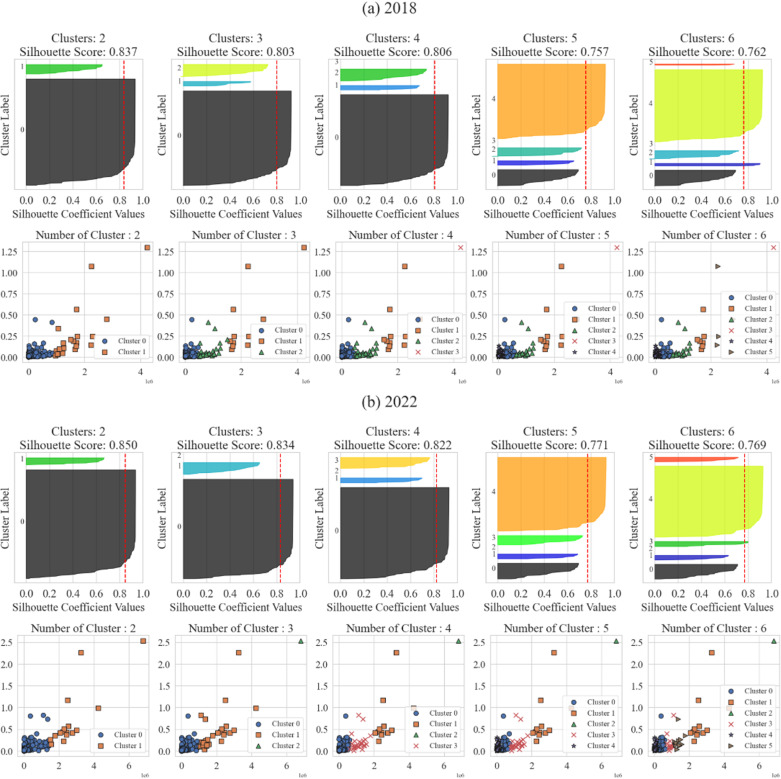
Clustering results (2018 and 2022). The silhouette scores indicate clustering quality based on the number of clusters (K). For the 2018 data, the highest silhouette score (0.837) occurs with two clusters (K =  2). As the number of clusters increases to three (0.803), four (0.806), five (0.757), and six (0.762), the scores decline. Similarly, in the 2022 data, the highest silhouette score (0.850) also occurs with two clusters, with subsequent scores for three (0.834), four (0.822), five (0.771), and six (0.769) clusters being lower.

Fig 4 presents the results of the clustering analysis along with corresponding silhouette scores, which assess the validity of cluster formations. The x-axis represents inter-regional land inequality, measured as land price per square meter, while the y-axis captures intra-regional inequality. Given the differing scales of these variables, standardization was applied to normalize values within a range of -1 to 1, ensuring accurate clustering outcomes.

Overall, the silhouette analysis for both years suggests that a two-cluster solution provides the best partitioning, offering the highest cohesion within clusters and separation between them.

Examining the optimal clustering outcomes presented in Table 2, Cluster 0 is comprised of 234 districts, while Cluster 1 contains 16 districts. Interestingly, the composition of each cluster remains constant between the years 2018 and 2022. In other words, across this five-year span, the total of 250 districts (si-gun-gu) consistently segregate into two distinct clusters: one containing 16 districts and the other comprising the remaining 234 districts. Notably, there is no alteration in cluster membership over this temporal window. The stability in cluster composition over the examined period reinforces the notion that the socioeconomic structure underlying land distribution is not only enduring but also verging on permanency. As elucidated in the previous subsection, this temporal persistence further emphasizes that land inequality is a deeply ingrained issue.

**Table 2:** Results by cluster and year.

	Cluster 0	Cluster 1
2018	234 districts	16 districts (Bundang-gu, Dongdaemun-gu, Dongjak-gu, Gangdong-gu, Gangnam-gu, Gwangjin-gu, Jongno-gu, Jung-gu, Mapo-gu, Seocho-gu, Seodaemun-gu, Seongdong-gu, Songpa-gu, Yangcheon-gu, Yeongdeungpo-gu, Yongsan-gu)
2022	234 districts	16 districts (Bundang-gu, Dongdaemun-gu, Dongjak-gu, Gangdong-gu, Gangnam-gu, Gwangjin-gu, Jongno-gu, Jung-gu, Mapo-gu, Seocho-gu, Seodaemun-gu, Seongdong-gu, Songpa-gu, Yangcheon-gu, Yeongdeungpo-gu, Yongsan-gu)

**Fig. 5:**
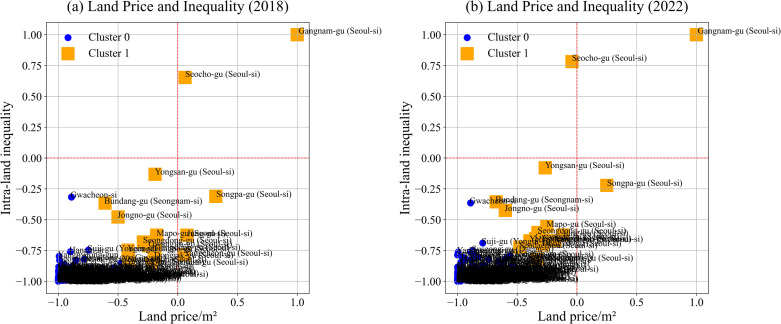
Optimal clusters. *Note*: ****K**** =  2 for both 2018 and 2022.

In scrutinizing the optimal clustering results for both 2018 and 2022 as depicted in Fig 5, several noteworthy observations emerge. The x-axis, representing land price per square meter, and the y-axis, indicating intra-land inequality, reveal distinct spatial relationships among districts. Specifically, Gangnam-gu in Seoul is prominently positioned in the upper right quadrant, while Seocho-gu in Seoul occupies a lower position. Most districts are tightly clustered in the lower left area, constituting what is identified as Cluster 0, whereas a smaller number of districts, constituting Cluster 1, are situated above those in Cluster 0. Remarkably, the overall structural characteristics of this clustering display minimal variation between the years 2018 and 2022. Furthermore, it is of interest to note that the districts within Cluster 1 exhibit a more dispersed distribution compared to those in Cluster 0. Such divergent clustering behaviors raise an intriguing question regarding disparities between districts in Cluster 1.

**Fig. 6:**
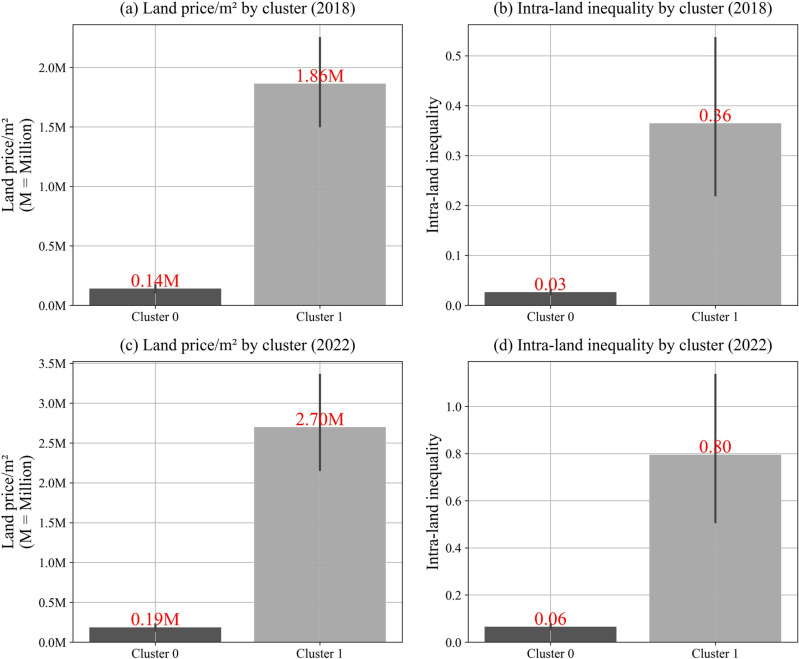
Gap of land price/m2 (KRW) and intra-land inequality by cluster (2018 and 2022).

Fig 6 offers a clear visualization of the disparities in average land price per square meter and intra-land inequality between the two clusters for 2018 and 2022. In 2018, as shown in panel (a), the average land price per square meter in Cluster 0 was 139,314 KRW, significantly lower than the 1,863,068 KRW observed in Cluster 1. This indicates that the land price in Cluster 1 was more than 13 times higher than in Cluster 0. By 2022, as displayed in panel (c), this disparity widened further, with Cluster 0’s average land price rising to 185,055 KRW, while Cluster 1’s average soared to 2,697,341 KRW, making it more than 14.5 times greater than Cluster 0.

This stark discrepancy highlights the uneven distribution of land prices across the clusters, reinforcing documented patterns of inequality while adding a temporal dimension to the analysis. The data suggest that the factors driving land price inflation are more concentrated and potent in Cluster 1. This cluster, characterized by premium residential and commercial zones, could be seen as a hotspot for real estate investments, likely benefiting from favorable public policies or capital inflows that contribute to its high land values, potentially at the expense of more marginalized areas [64].

Panel (b) of Fig 6 shows significant differences in intra-regional land inequality between the clusters in 2018. Cluster 0 had a low average ratio of 0.03, indicating that most households in these districts owned land assets valued at less than 50 million KRW, with very few owning assets exceeding 1 billion KRW. This reflects relatively low levels of land ownership inequality. In contrast, Cluster 1 had a much higher ratio of 0.36, suggesting that about 36% of households with land assets below 50 million KRW were counterbalanced by a significant proportion of households owning assets valued at over 1 billion KRW, signaling much higher inequality in land ownership.

By 2022, intra-regional land inequality had worsened, as depicted in panel (d). The average ratio for Cluster 0 rose slightly to 0.06, while Cluster 1’s ratio surged to 0.8. This sharp increase in Cluster 1 reflects a growing concentration of land wealth, where households owning high-value land assets are increasingly more prevalent. The widening gap between the clusters, particularly the pronounced increase in Cluster 1, suggests that land inequality is driven by deeper systemic or localized economic and social forces rather than random variation.

**Fig 7:**
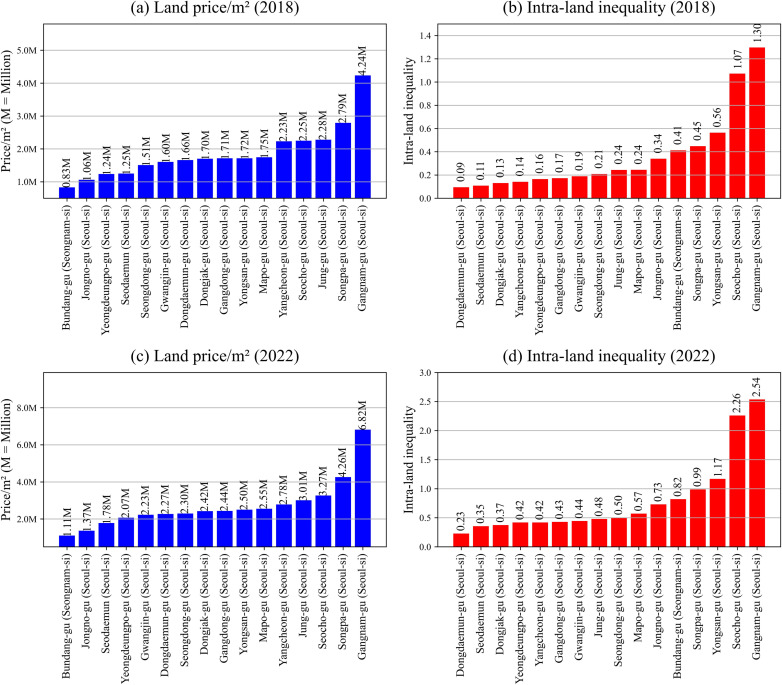
Land price/m2 (KRW) and intra-land inequality in Cluster 1. Moreover, as previously shown in Fig 5, the districts in Cluster 1 exhibited greater variation in land prices compared to those in Cluster 0. Fig 10 confirms this trend, with Cluster 1 displaying higher variance. This suggests that not only are the districts in Cluster 1 distinct from those in Cluster 0, but there is also significant internal disparity within Cluster 1 itself. These findings point to the need for further analysis to better understand the factors driving intra-cluster inequality within Cluster 1.

Fig 7 serves as a graphical representation illuminating the disparities both in per square meter land prices and intra-regional land inequality within the districts comprising Cluster 1. The figure underscores a stark divergence among these districts, both in terms of asset value per square meter and wealth distribution among households. An examination of subfigures (a) and (c) in Fig 7 reveals a conspicuous divergence in land values among different regions. Gangnam-gu is markedly the highest, with a substantial gap separating it from other regions. Specifically, in 2018, there is a difference of approximately 1,500,000 KRW between Gangnam-gu and Songpa-gu, the second-highest region. Furthermore, this value is nearly fivefold that of Bundang-gu, the region with the lowest land values. By 2022, this chasm has widened further; the difference between Gangnam-gu and Songpa-gu escalates to about 2,600,000 KRW, roughly six times greater than that of Bundang-gu.

Turning our attention to subfigures (b) and (d) in Fig 7, we find that Gangnam-gu also leads in intra-regional land inequality, followed closely by Seocho-gu. Comparing the data from 2018 and 2022, there is a discernible increase in inequality levels. Notably, these two districts outpace others significantly in this aspect. In 2022, Gangnam-gu and Seocho-gu each have more than twice as many households possessing land assets greater than 1 billion KRW as those with assets valued at less than 50 million KRW. This suggests that these districts are increasingly becoming enclaves of concentrated wealth, further exacerbating regional disparities in land ownership.

Overall, this study reveals a pattern of land inequality in Korea, marked by disparities across geographic scales and an intensification from 2018 to 2022. The findings emphasize stark regional differences in land values, with the Seoul metropolitan area consistently showing much higher prices compared to other regions, reinforcing Seoul’s central role in land economics and the structural nature of these inequalities. Within Seoul, significant intra-urban disparities are evident, particularly in the Gangnam 3-gu districts, known for their exceptionally high land values and socioeconomic distinction. This highlights a sharp wealth divide in the capital. In the broader Seoul metropolitan area, districts such as Gangnam 3-gu, Yongsan-gu, and Jongno-gu further illustrate intra-regional inequality with concentrated wealth.

Cluster analysis identifies two primary groups, with the first comprising 16 districts characterized by significantly higher land prices and greater inequality than the second cluster. The gap between these clusters widened from 2018 to 2022, with wealth concentration intensifying in districts like Gangnam-gu and Seocho-gu. This suggests distinct forces exacerbating inequality even within affluent areas.

## Conclusion

This study set out to examine the evolution of land inequality in Korea, particularly in the Seoul Metropolitan Area, from 2018 to 2022. By employing a data-driven approach that integrates land inequality metrics and machine learning techniques, this study identifies significant inter-regional and intra-regional disparities, with specific districts—most notably Gangnam-gu and Seocho-gu—emerging as focal points of wealth concentration. The findings highlight a deepening pattern of inequality, reinforcing the argument that land-based disparities in Korea are not transient fluctuations but rather systemic and intensifying structural issues.

One of the key limitations of this study is the constrained temporal scope of the available district-level data, which enables an analysis of land inequality over a relatively short period but does not provide a long-term historical perspective. While the study offers important insights into the evolution of land inequality from 2018 to 2022, the lack of extended time-series data restricts the ability to determine whether the observed patterns reflect cyclical fluctuations, a continuation of pre-existing trends, or a more recent structural transformation. A more comprehensive historical analysis would enhance the understanding of how land inequality has developed over time and its broader socioeconomic implications.

Future research could address this limitation by extending the temporal scope of the dataset to capture longer-term trends. This may necessitate the digitization of archival records that have not yet been systematically compiled, allowing for a more robust historical analysis of land inequality. Expanding the dataset in this manner would facilitate a deeper examination of the structural forces shaping land distribution and provide stronger empirical foundations for assessing policy interventions aimed at mitigating land-based disparities.

While clustering techniques provide valuable insights into structural patterns of land inequality, their interpretability requires caution. As noted in the literature [65], clustering outcomes are sensitive to methodological choices, including algorithm selection, distance metrics, and the number of clusters. Although K-means++ improves centroid initialization, its assumption of spherical clusters may not fully capture the spatial complexity of land inequality. Moreover, the identified clusters are based on inter-regional land price disparities and intra-regional wealth concentration, which, while meaningful, do not account for broader economic and policy dynamics. Therefore, the clusters should be viewed as indicative rather than definitive classifications. Future research could enhance robustness by incorporating additional spatial and economic variables or exploring alternative clustering methods to refine the analysis of land inequality’s evolving structure.

In contemporary society, real estate functions as a positional good, where ownership signifies economic privilege and social distinction. As discussed in the theoretical framework, landownership extends beyond its financial role, serving as a key mechanism for reproducing socioeconomic inequality. This dynamic enables landowners to consolidate economic power while creating systemic barriers that limit access to high-value property markets for non-owners. The concentration of land wealth in prime locations not only drives up property values but also reinforces exclusionary patterns, restricting access to housing, quality education, and employment opportunities. Thus, land inequality is not merely a reflection of disparities in wealth but a broader indicator of structural socioeconomic inequality, where access to essential resources and opportunities is increasingly determined by landholding status.

The implications of these findings are significant. The growing disparities in landownership and valuation pose challenges to both economic equity and social cohesion, raising concerns about long-term political and social stability. The concentration of land wealth in a limited number of districts parallels historical patterns of land monopolization, highlighting the risks of deepening socioeconomic stratification. Without targeted policy interventions, these disparities are likely to exacerbate inequalities in housing accessibility, educational opportunities, and financial security, further entrenching social divisions.

The broader relevance of this study extends beyond Korea, aligning with the challenges faced by other rapidly urbanizing economies, particularly within the developmental state model prevalent in Asia. Korea’s experience illustrates the unintended consequences of policy-driven urban concentration, underscoring the risks associated with unchecked land speculation and wealth accumulation. These insights are valuable for policymakers navigating similar urbanization trajectories and land market dynamics in other contexts.

Addressing land inequality requires a multi-faceted policy approach. In addition to reassessing Seoul-centric development policies, there is a critical need to consider more equitable land taxation frameworks and housing affordability measures. The findings also highlight the importance of addressing intra-regional disparities, particularly within affluent districts, through policies that balance economic growth with spatial equity. Furthermore, given the interplay between land wealth and political power, reform efforts must engage with broader questions of democratic governance and economic justice, ensuring that policies do not disproportionately benefit landowners at the expense of broader societal welfare.

Ultimately, this study highlights the urgency of addressing land inequality as a fundamental socioeconomic challenge. By exposing the entrenched nature of these disparities, it contributes to ongoing discussions on sustainable urban development and economic justice, aligning with global development goals such as SDG 10 (Reduced Inequalities) and SDG 11 (Sustainable Cities and Communities). As Korea and other rapidly urbanizing economies confront the issue of land inequality, comprehensive and long-term policy interventions will be necessary to promote a more equitable and sustainable urban future.

## Supporting information

S1 Appendix Data information and descriptive statistics. (PDF)
