# Supplementary material for: Mapping the landscape of land inequality: A multi-level, data-driven exploration of land inequality in South Korea’s urban and regional spheres
Source: PLoS One. 2025 Mar 25;20(3):e0320252. doi: 10.1371/journal.pone.0320252 (PMC11936173; doi:10.1371/journal.pone.0320252)
Supplement: S1 Appendix — (PDF) [file pone.0320252.s001.pdf]

Mapping the Landscape of Land Inequality: A Multi-Level, Data-Driven Exploration of Land Inequality in South Korea's Urban and Regional Spheres

Online Appendix

Tables

Table A1. Data information ... 2
Table A2. Descriptive statistics, pre-standardization (2018) ... 3
Table A3. Descriptive statistics, pre-standardization (2022) ... 3
Table A4. Descriptive statistics, post-standardization (2018) ... 3
Table A5. Descriptive statistics, post-standardization (2022) ... 3

Appendix A

Table A1. Data information

	Data	Source	Website
1	Land price	Ministry of Land, Infrastructure and Transport	https://stat.molit.go.kr/portal/cate/statView.do?hRsId=25&hFormId=6231&hSelectId=6222&hPoint=0&hAppr=1&hDivEng=&oFileName=&rFileName=&midpath=&sFormId=6231&sStart=2022&sEnd=2022&sStyleNum=1&settingRadio=xlsx
2	Gross area (m^2) by district (si-gun-gu)	Statistics Korea	https://kosis.kr/statHtml/statHtml.do?orgId=116&tblId=DT_MLTM_2300&conn_path=I3
3	Intra-land inequality	Ministry of Land, Infrastructure and Transport	https://stat.molit.go.kr/portal/cate/statView.do?hRsId=25&hFormId=6222&hSelectId=6231&hPoint=0&hAppr=1&hDivEng=&oFileName=&rFileName=&midpath=&sFormId=6222&sStart=2022&sEnd=2022&sStyleNum=1&settingRadio=xlsx

Table A2. Descriptive statistics, pre-standardization (2018)

	N	Mean	Std dev	Min	Median	Max
Land price/m^2	250	249634.4	522375.2	758.78	28963.4	4235819
Intra-land inequality	250	0.047	0.124	0.001	0.015	1.297

Table A3. Descriptive statistics, pre-standardization (2022)

	N	Mean	Std dev	Min	Median	Max
Land price/m^2	250	345841.8	767747.9	1055	35073.6	6817358
Intra-land inequality	250	0.111	0.257	0.003	0.035	2.535

Table A4. Descriptive statistics, post-standardization (2018)

	N	Mean	Std dev	Min	Median	Max
Land price/m^2	250	-0.882	0.246	-1	-0.986	1
Intra-land inequality	250	-0.929	0.192	-1	-0.978	1

Table A5. Descriptive statistics, post-standardization (2022)

	N	Mean	Std dev	Min	Median	Max
Land price/m^2	250	-0.898	0.225	-1	-0.99	1
Intra-land inequality	250	-0.914	0.203	-1	-0.974	1
